# Associations between exercise motivation and self-efficacy through leisure satisfaction and psychological resilience in college students: a PLS-SEM study

**DOI:** 10.3389/fpubh.2026.1763971

**Published:** 2026-04-29

**Authors:** Taiping Li, Qiuxian Ye, Yafei Yuan, Jifeng Dong, Yanqing Yan

**Affiliations:** 1 Guangdong University of Science and Technology, Dongguan, Guangdong, China; 2 Zhuhai College of Science and Technology, Zhuhai, Guangdong, China; 3 Army Special Operations College, Guilin, Guangxi, China

**Keywords:** exercise motivation, leisure satisfaction, mood disorders, psychological resilience, self-efficacy, young adults

## Abstract

**Objective:**

Growing concern has emerged regarding the mental health of young adults, particularly within university settings where academic and psychosocial pressures are prominent. Exercise motivation (EM), conceptualized within self-determination theory (SDT), especially intrinsic and identified regulation, has been associated with psychological well-being. However, the structural associations among exercise motivation (EM), leisure satisfaction (LS), psychological resilience (PR), and self-efficacy (SE) in young adults remain insufficiently clarified.

**Methods:**

This cross-sectional study examined these associations in a sample of 437 university students aged 18–23 years in China. Data were collected through validated self-report instruments and analyzed using partial least squares structural equation modeling (PLS-SEM).

**Results:**

The results indicated that exercise motivation was positively associated with leisure satisfaction (β = 0.794, *p* < 0.001), leisure satisfaction was positively associated with psychological resilience (β = 0.342, *p* < 0.001), and PR was positively associated with self-efficacy (β = 0.228, *p* < 0.001). Sequential mediation analysis showed that leisure satisfaction and PR jointly accounted for a significant indirect association between EM and SE (β = 0.062, *p* = 0.001). Model fit indices (*SRMR* = 0.055; *NFI* = 0.847) indicated an acceptable level of fit within the prediction-oriented PLS-SEM approach.

**Conclusion:**

These findings indicate that exercise motivation is statistically associated with psychological resources among young adults. Although causal inferences cannot be drawn due to the cross-sectional design, the study contributes to understanding motivational processes within the SDT framework and provides directions for future longitudinal research.

## 1 Introduction

Adolescence is a key developmental stage characterized by physical, emotional and cognitive changes. During this developmental period, the prevalence of mood disorders has shown increasing trends in several regions, contributing substantially to the global burden of disease (1, 2). Adolescent depression (3) is a major public health problem of global concern. The WHO identifies depression as a primary contributor to mood disorders in young adults globally. Epidemiological evidence suggests that approximately 10%−20% of adolescents report depressive symptoms at clinically relevant levels, based on large-scale population studies (4). In China, the escalation of social competitiveness, the rise in pressure from academia, and the transformation of family structure (5), the incidence of adolescent depression shows an obvious upward trend. This trend has attracted wide attention in the field of public health (6). For example, a longitudinal study of Chinese college students found that 20–40 % of college students experienced varying degrees of depression, anxiety and stress, and about 35 % of college students showed higher levels of depression than the general population (7). Previous studies have identified multiple psychosocial stressors associated with increased depressive symptoms among college students (8, 9). The onset of major depressive disorder and related mood disorders mostly occurs in adolescence. Common comorbidities include non-suicidal self-injury, obsessive-compulsive disorder, and hyperactivity. These conditions not only damage academic performance and interpersonal relationships, but also make individuals vulnerable to chronic mental illness in adulthood. Therefore, identifying protective psychological factors that are associated with adaptive functioning and emotional stability is crucial for achieving sustainable mental health in young adults.

In recent years, exercise has been widely recognized as a non-pharmacological approach associated with improved mood-related outcomes among adolescents (10, 11). Relevant studies have shown that regular exercise has been associated with improved mood status, lower levels of depressive symptoms, and higher self-regulation (12). However, previous research has mainly focused on the frequency or intensity of exercise (13, 14), with limited exploration of the intrinsic ways in which exercise is related to mental health through psychological mechanisms. Deepening the understanding of how exercise motivation is transformed into psychological resilience (PR) and self-efficacy (SE) can reveal the underlying mechanisms by which healthy behaviors affect mental health.

The self-determination theory (SDT) provides a solid theoretical foundation for exploring this issue. In this study, exercise motivation mainly reflects intrinsic and identified regulation, representing a relatively autonomous form of motivation in the SDT continuum. The theory holds that individuals engaged in sports driven by intrinsic motivation such as interest, enjoyment or personal growth are more likely to obtain psychological satisfaction and positive emotional experience from activities (15). This motivational orientation satisfies three basic mental needs—autonomy, competence and relationality—and thus promotes subjective well-being and resilience (16, 17). At the same time, from the perspective of positive psychology, positive leisure experience can broaden the individual's cognitive vision and accumulate psychological resources, thereby being associated with higher coping ability and self-efficacy. In this process, leisure satisfaction (LS) (18), as an intermediary variable at the emotional level, links exercise motivation with psychological resilience. When young adults get pleasure and satisfaction from sports, they are more likely to maintain emotional stability, enhance psychological resilience, and is associated with greater adaptability (19, 20). Young adults with higher resilience tend to show stronger optimism, adaptability and resilience, thereby reducing the risk of depression and anxiety. Psychological resilience is positively associated with self-efficacy, reflecting individuals' beliefs in their ability to regulate emotions, meet challenges, and achieve goals (12). Higher self-efficacy can not only enhance the ability to solve problems, but also effectively buffer stress (21).

Although existing research supports the link between exercise and mental health, there is still a lack of empirical evidence on the sequential mediating mechanism of exercise motivation affecting SE through leisure experience and psychological resilience. In order to solve this research gap, this study uses the structural equation model to systematically test the relationship between EM, LS, psychological resilience and SE. By clarifying these psychological associations, this study aims to contribute theoretical insight and inform the development of non-pharmacological mental health promotion strategies among young adults, while offering reference for school-based mental health education.

## 2 Literature review and research hypotheses

Motivation is an intrinsic psychological tendency, which initiates and maintains individual behavior, and at the same time directs behavior to specific goals, which is the intrinsic decisive force of behavior (22). Among them, intrinsic motivation is particularly critical. Motivations that directly affect individuals' behavioral choices can be classified as internal or external influences that encourage involvement in leisure sports activities (23). It may come from personal needs, aspirations, and values; it may also be influenced by external variables, such as incentives, pressure from society, or the expectations of other people (24). Multiple motivations such as achievement pursuit, health appeal, and social needs, will significantly affect the engagement, persistence, and performance level of individuals (20).

The satisfaction gained from participating in exercise leisure activities can be understood as the positive emotions aroused by actively choosing and participating in such activities (19, 25), which reflects the individual's satisfaction with their own leisure experience or situation. Sports and leisure sports clearly play a key role in shaping college students' self-awareness and maintaining mental health. These activities augment physical vitality, broaden social relationships, and foster psychological development (20).

Psychological resilience can be regarded as an individual's innate endowment in the context of competition and high pressure and can also be regarded as an acquired skill, which helps people to cope with the accompanying stress and anxiety continuously and effectively (26, 27). The concept refers to the capacity to sustain resolve, self-assurance, and a sense of agency throughout challenges. Its structure is hierarchical and multidimensional, but research is still scarce compared to hot topics such as goals, emotions, and cognition (28). As a dynamic process, resilience is shaped by multiple factors, include optimism, resilience, and self-improvement (29). The improvement of resilience level not only helps to maintain mental health but also promotes the development of healthy behaviors and positive lifestyles in some situations (21).

General SE pertains to a person's assessment and conviction that they can make plans, initiate actions, and achieve goals. This structure regulates behavior through the level of self-confidence. Physical SE emphasizes the person's assurance in the robustness of physical fitness required to participate in sports and physical activity (30). The exercise self-efficacy of young women has been reported to be associated with the frequency and intensity of formal training, and these indicators have been reported to be positively associated with health outcomes (31). Recent evidence further points out that adolescent SE is positively correlated with physical activity participation (32). Exercise and SE are significantly positively correlated: high self-efficacy is more confident and calm in the face of risks and challenges (21).

**Figure 1:**
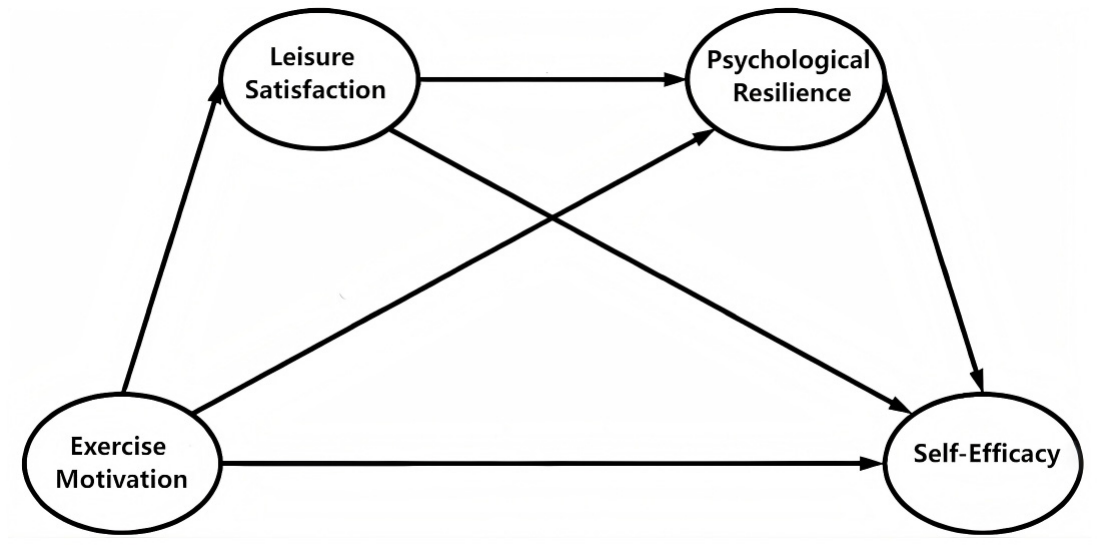
Hypothesized model.

Based on SDT (15), this study explains how exercise motivation affects young adults' mental health along the “basic psychological needs satisfaction-motivation quality-adaptation function.” SDT believes that the degree of satisfaction of the three needs of autonomy, competence and relationality in the activity determines the depth and stability of motivation from external regulation to internal motivation internalization. In the context of this study, when young adults are engaged in sports due to interest, fun, or self-growth, it is easier to obtain needs satisfaction in the process, accompanied by positive emotions and value judgments, which is manifested as higher satisfaction with leisure sports experience. From the perspective of SDT, these positive feedbacks catalyzed by high-quality motivation will strengthen competence, and autonomy and then is associated with higher self-efficacy; continuing need satisfaction and active engagement also help individuals form adaptive adjustment and “rebound” ability to stress, that is, psychological resilience. Therefore, based on the literature review and proposed hypotheses, this study constructs a theoretical hypothesis model as shown in Figure 1 and proposes the following five hypotheses:

H1: Exercise motivation is positively associated with leisure satisfaction among young adults.
H2: Leisure satisfaction is positively associated with psychological resilience.
H3: Psychological resilience is positively associated with self-efficacy.
H4: Leisure satisfaction is indirectly associated with self-efficacy through psychological resilience.
H5: Exercise motivation is indirectly associated with self-efficacy through the sequential associations of leisure satisfaction and psychological resilience.

## 3 Methods

### 3.1 Participants

In this study, convenience sampling was employed to recruit college students enrolled in universities in Guangdong Province, China. The questionnaire was distributed electronically via an online survey platform between September and October 2025. The data collection period corresponds to the beginning of the autumn semester in Chinese universities, during regular teaching weeks rather than exam periods. Therefore, participants engaged in normal academic activities during the survey. With the assistance of faculty and staff, the survey link was shared through university class groups and student social media networks. Participation is voluntary and anonymous. A total of 454 questionnaires were collected. Data filtering was conducted before analysis.

Inclusion criteria were as follows: (1) currently enrolled as a full-time undergraduate student in a university in Guangdong Province; (2) aged between 18 and 23 years; (3) able to understand and complete the online questionnaire independently; and (4) provided informed consent to participate.

Exclusion criteria included: (1) self-reported diagnosis of severe psychiatric disorders that might impair the ability to complete the questionnaire; (2) incomplete questionnaire submission; and (3) failure to meet data quality standards (e.g., response time shorter than 120 s, more than 10% missing responses, or patterned response behavior).

Based on these criteria, 17 questionnaires were excluded. The final valid sample consisted of 437 participants, yielding a valid response rate of 96.25%. Participants included 207 males (47.37%) and 230 females (52.63%); 270 participants aged 18–20 (61.8%), 165 aged 21–22 (37.7%), and only 3 aged 23–24; humanities majors: 162 (37.1%); science and engineering majors: 182 (41.6%); and arts majors: 93 (21.3%).

### 3.2 Measures

All scales were administered in Chinese. When the original instruments were not available in Chinese, a standard translation and back-translation procedure was applied following Brislin's guidelines. First, two bilingual researchers independently translated the items into Chinese. A separate bilingual expert then back-translated the Chinese version into English. Discrepancies were discussed and resolved to ensure semantic equivalence.

Previous studies have reported acceptable reliability and validity of these scales in Chinese samples. In the present study, construct validity was further evaluated through PLS-SEM measurement model assessment, including factor loadings, composite reliability (CR), average variance extracted (AVE), and discriminant validity (HTMT and Fornell–Larcker criteria). The results demonstrated satisfactory psychometric properties within the current sample.

#### 3.2.1 Exercise motivation

EM was measured using the 5-item scale developed by Biese et al. (33), reflecting intrinsic and identified regulation. The scale is unidimensional in the present study. A sample item is: “Because I love the feeling of being totally immersed in a sport.” Responses were rated on a 5-point Likert scale (1 = strongly disagree, 5 = strongly agree). In the current sample, Cronbach's α was 0.897.

#### 3.2.2 Leisure satisfaction

LS was assessed using the 9-item short form of the Leisure Satisfaction Scale developed by Trottier et al. (34). The scale is treated as a unidimensional construct in this study. A sample item is: “Participating in leisure sports activities has boosted my confidence.” In the present sample, Cronbach's α was 0.954.

#### 3.2.3 Psychological resilience

PR was measured using the 7-item Adolescent Resilience Scale developed by Oshio et al. (35). The scale was modeled as a unidimensional construct. A sample item is: “In the presence of difficulties, I focus all my energy on overcoming them.” Cronbach's α in the current sample was 0.884.

#### 3.2.4 Self-efficacy

SE was assessed using the 7-item version of the General Self-Efficacy Scale adapted by Steigen et al. (36). The scale was treated as a single-factor construct. A sample item is: “I can face difficulties with equanimity because I believe in my ability to handle things.” Cronbach's α was 0.921.

### 3.3 Procedure

In this study, we used the Exercise Motivation, Leisure Satisfaction, Psychological Resilience, and Self-Efficacy scales to collect data through online questionnaires. All subjects agreed to sign the informed consent form before starting to fill out the online questionnaire. After the participants completed all the items, the test data was automatically generated. The procedure was approved by the Ethics Committee of Guangdong University of Science and Technology.

### 3.4 Statistical analysis

This study uses SPSS 29.0 software for descriptive statistics on the data. Additionally, we will use SmartPLS 4.1 software for the measurement model and structural evaluation during the data analysis process. This work applies PLS-SEM. This survey chose this software for analysis because it has been successful in evaluating validity and reliability and confirming or rejecting hypotheses (37). In terms of analytical advantages, partial least squares can simultaneously estimate the path coefficients of the specified model and the loadings of individual items. Consequently, it enables researchers to circumvent biased and inconsistent parameter estimates (38) and is applied to more advanced and complex models (39).

A priori power analysis was conducted using G^*^Power 3.1 to determine the minimum required sample size for detecting a medium effect size (*f*^2^ = 0.15) at a significance level of 0.05 and statistical power of 0.95. The analysis indicated that a minimum sample size of 107 participants was required for a model with up to two predictors. The final sample size of 437 therefore exceeded the recommended threshold, ensuring adequate statistical power to detect the hypothesized associations.

Additionally, according to the “10-times rule” commonly applied in PLS-SEM research, the minimum sample size should be 10 times the maximum number of structural paths directed at a particular construct. As the most complex construct in the model received two incoming paths, the required minimum sample size was 20. The current sample size substantially exceeds this guideline.

## 4 Results

### 4.1 Common method bias test

All variables in this study were measured through self-report questionnaires from the same respondents. To reduce the potential common method bias, the study adopted several procedural control measures during the data collection process. First, at the beginning of the questionnaire, the respondents were clearly informed that the study was only for academic purposes. All responses will be maintained in full confidentiality, and there are no “correct” or “incorrect” answers, so as to reduce the social expectation effect. At the same time, respondents were encouraged to answer based on their true feelings, so as to improve the authenticity and reliability of the data. In addition, the presentation order of the questionnaire items was randomized to reduce the systematic bias that may be caused by the order of answers (40). In terms of statistical control, the common method bias was tested using the full collinearity test in SmartPLS software based on Kock's suggestion (41). The results indicated that the variance inflation factor (VIF) values for all latent constructs were below the threshold of 3.3 (42), indicating that there is no significant common method bias in this study.

### 4.2 Measurement model

According to Table 1, all constructs demonstrated acceptable reliability and convergent validity. Specifically, Cronbach's α values ranged from 0.846 to 0.945, and CR values ranged from 0.884 to 0.954, both exceeding the recommended threshold of 0.70. AVE values were between 0.551 and 0.696, higher than the minimum cutoff of 0.50, indicating satisfactory convergent validity. In addition, according to the standard, item factor loadings should exceed 0.7 (43). Although factor loadings for some items fell between 0.60 and 0.70, the CR of each latent variable exceeded 0.70, and the AVE surpassed 0.50. Consequently, the overall convergent validity remains satisfactory (44). The HTMT values in Table 2 ranged from 0.723 to 0.849, which are all below the conservative threshold of 0.85. This demonstrates that each construct is empirically distinct and meets the requirements of discriminant validity (43). As indicated in Table 3, the square roots of the AVEs (diagonal values) were all greater than the inter-construct correlations (off-diagonal values). These results further confirm the discriminant validity of the measurement model (43).

**Table 1:** Reliability and validity.

Constructs	Items	Outer loadings	Cronbach' α	CR	AVE
Exercise motivation	EM 1	0.814	0.856	0.897	0.635
	EM 2	0.780			
	EM 3	0.759			
	EM 4	0.859			
	EM 5	0.768			
Leisure satisfaction	LS 1	0.841	0.945	0.954	0.696
	LS 2	0.853			
	LS 3	0.848			
	LS 4	0.850			
	LS 5	0.816			
	LS 6	0.837			
	LS 7	0.833			
	LS 8	0.821			
	LS 9	0.810			
Psychological resilience	PR 1	0.691	0.846	0.884	0.551
	PR 2	0.713			
	PR 3	0.770			
	PR 4	0.790			
	PR 5	0.714			
	PR 6	0.702			
	PR 7	0.664			
Self-efficacy	SE 1	0.670	0.899	0.921	0.625
	SE 2	0.799			
	SE 3	0.820			
	SE 4	0.796			
	SE 5	0.807			
	SE 6	0.795			
	SE 7	0.837			

**Table 2:** Discriminant validity (HTMT criterion).

Constructs	EM	LS	PR	SE
EM				
LS	0.849			
PR	0.721	0.684		
SE	0.810	0.806	0.723	

**Table 3:** Discriminant validity (Fornell–Larcker criterion).

Constructs	EM	LS	PR	SE
EM	**0.807**			
LS	0.794	**0.834**		
PR	0.612	0.612	**0.722**	
SE	0.717	0.749	0.633	**0.791**

Bold values on the diagonal represent the square root of the average variance extracted (AVE) for each construct.

### 4.3 Structural model

The VIF values were examined to assess potential collinearity issues among the constructs. As shown in Table 4, the VIF values for EM, LS, PR, and SE ranged between 1.000 and 2.903. All values were well below the commonly accepted threshold of 3.3, indicating that multicollinearity was not a concern in the structural model. This result confirms that the predictor variables were sufficiently independent and suitable for subsequent path analysis (43, 45).

**Table 4:** Collinearity test.

Constructs	EM	LS	PR	SE
EM		1.000	2.701	2.900
LS			2.701	2.903
PR				1.718
SE				

The PLS guidance technique with a sample size of 5,000 was used to evaluate the size and importance of the model path coefficient (45). Table 5 summarizes the test results. In the structural model, the significance test aims to determine the influence of exogenous variables on endogenous variables.

**Table 5:** Path hypothesis testing.

Hypothesis	Original sample (β)	2.50%	97.50%	*P*	Results
EM → LS	0.794	0.743	0.841	0.000	Support
LS → PR	0.342	0.215	0.475	0.000	Support
PR → SE	0.228	0.120	0.333	0.000	Support

**Figure 2:**
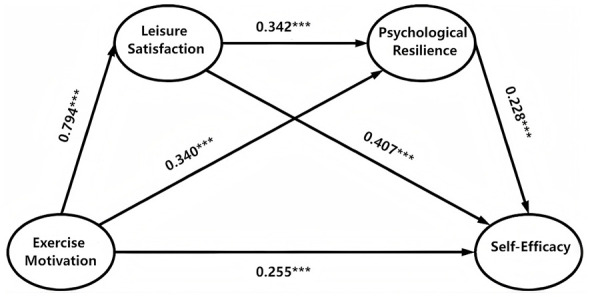
The structural equation model illustrates the relationships among exercise motivation, leisure satisfaction, psychological resilience, and self-efficacy. ***P < 0.001.

The structural model was evaluated to test the hypothetical relationship between exercise motivation, leisure satisfaction, psychological resilience and SE. As shown in Table 5 and Figure 2, the direct paths of all hypotheses are positive and statistically significant. Specifically, exercise motivation was significantly associated with leisure satisfaction (β = 0.794, *p* < 0.001), indicating that young adults with higher exercise intrinsic motivation experienced higher satisfaction in leisure activities. Similarly, leisure satisfaction was significantly associated with psychological resilience (β = 0.342, *p* < 0.001), indicating that young adults who feel more satisfied and satisfied in the leisure experience can better develop adaptive coping mechanisms and emotional stability. Finally, psychological resilience was significantly associated with SE (β = 0.228, *p* < 0.001), indicating that young adults with higher resilience also showed higher confidence in coping with challenges and achieving personal goals.

The *R*^2^ value (see Table 6) shows that the model has good explanatory power. The predictor variance accounted for 63.0 % of the LS variance, 41.8 % of the PR variance, and 63.2 % of the SE variance. In addition, all *Q*^2^ values exceeded zero, indicating a strong predictive correlation. This shows that the model not only has the ability to explain, but also has a reliable predictive ability for the data. To evaluate model fit, SRMR and NFI were reported. The SRMR value was 0.055, which is below the recommended threshold of 0.08, indicating an acceptable level of residual discrepancy. The NFI value was 0.847, which is below the conventional 0.90 benchmark commonly applied in covariance-based SEM. However, in PLS-SEM research, NFI values above 0.80 are generally considered acceptable for exploratory and prediction-oriented models (43). Therefore, the model fit indices should be interpreted cautiously. Taken together, the results suggest that the structural model demonstrates an acceptable level of fit within the context of PLS-SEM. It is important to note that PLS-SEM primarily emphasizes predictive accuracy rather than global goodness-of-fit measures (see Table 6).

**Table 6:** Explanatory power and predictive relevance.

Constructs	*R* ^2^	*Q* ^2^	Model fit
LS	0.630	0.626	SRMR: 0.055
PR	0.418	0.365	NFI: 0.847
SE	0.632	0.511	

### 4.4 Mediation analysis

Both intermediary paths are identified as partial mediations (see Table 7). Specifically, psychological resilience partially mediated the relationship between leisure satisfaction and self-efficacy (β = 0.078, *p* = 0.001), indicating that young adults' satisfaction with leisure experience directly and indirectly is related to SE through PR.

**Table 7:** Mediating analysis.

Relationship	Indirect effect	2.5%	97.5%	*t*	*P*	Direct effect	Type of mediation
LS → PR → SE	0.078	0.037	0.128	3.378	0.001	0.407	Partial mediation
EM → LS → PR → SE	0.062	0.030	0.102	3.338	0.001	0.255	Partial mediation

In addition, EM has a partial sequential mediation effect on SE through LS and PR (β = 0.062, *p* = 0.001), indicating that young adults with exercise motivation experience greater LS and PR, which together are associated with stronger beliefs in personal efficacy.

## 5 Discussion

Grounded in SDT, this study integrates perspectives from motivation and positive psychology to examine the structural associations among exercise motivation, leisure satisfaction, psychological resilience, and self-efficacy in young adults. SDT posits that autonomous forms of motivation—such as intrinsic interest and identified regulation—facilitate the satisfaction of basic psychological needs for autonomy, competence, and relatedness, thereby fostering adaptive psychological functioning. Consistent with this framework, the findings indicate that exercise motivation is positively associated with leisure satisfaction. When young adults engage in physical activity driven by intrinsic interest or personal value, they are more likely to experience enjoyment, growth, and a sense of belonging during participation (15). These positive experiential outcomes may enhance satisfaction with leisure activities, which in turn serves as an important psychological resource. The present study aims to clarify this motivational pathway and provide a theoretically informed explanation of how exercise-related experiences are associated with indicators of mental well-being among young adults (20).

Secondly, the study shows that leisure satisfaction was positively associated with psychological resilience: positive emotions broaden young adults' cognitive and coping paths and accumulate lasting psychological resources for them. When they gain positive experience in sports or other leisure activities, stress and anxiety may be lower, and their ability to self-regulate and cope with difficulties is enhanced simultaneously, which may be reflected in higher levels of psychological resilience (46). Therefore, a positive leisure experience is not a short-term emotional compensation but an important source of long-term accumulation of PR, which is consistent with the findings of Shin and You (47).

This study once again confirms that psychological resilience was positively associated with self-efficacy, which is fully consistent with the SDT proposed by Deci and Ryan, which emphasizes the autonomy of individual behavior and its motivation process (48). Bandura pointed out that successful coping experiences and positive feedback can shape the belief that “I can do it” and then improve self-efficacy (49). Young adults with high resilience show stronger confidence and sense of control in stressful situations and can regulate emotions and adhere to goal-oriented behavior (50, 51). This result is also consistent with Peng et al.'s (12) finding that resilience is an important resource for adolescent mental health and contributes to the development of positive psychological quality.

Finally, the study further found that LS and PR constitute a chain intermediary between EM and SE. This path outlines the evolution process of “motivation activation-obtaining positive experience-accumulating psychological resources-forming ability beliefs.” Specifically, when young adults are engaged in sports due to their intrinsic interests, the pleasure and satisfaction brought by the activities themselves are associated with higher leisure satisfaction. This positive experience then contributes to higher psychological resilience, which is associated with higher self-efficacy. The research findings integrate self-determination theory and explain how intrinsic motivation is transformed into adaptability and confidence through positive emotions, thereby forming a dynamic chain associated with better mental health outcomes. It further elucidates the relationship between exercise and mental health, and helps to understand the psychological resources related to emotional health.

In addition to the SDT based interpretation, other interpretations should also be considered. Considering the cross-sectional design, young people with high baseline self-efficacy or PR are more likely to participate in exercise with autonomous motivation, not just the motivation before psychological adaptation. In addition, unmeasured situational variables, including social support and personality traits (such as optimism) or previous exercise habits, may simultaneously affect motivation orientation and psychological resources. Therefore, although the current research results are theoretically consistent with SDT, explanations for the reciprocal or third variable cannot be ruled out. Future longitudinal and experimental research needs to uncover these potential pathways.

### 5.1 Theoretical implications

Importantly, this study extends previous research on the SE pathway in young people by emphasizing the crucial role of PR. Although previous studies have examined the relationship between EM and SE or between PR and MH outcomes (12, 21), few studies have designated resilience as a continuous psychological mechanism linking experience satisfaction with efficacy beliefs. The research findings indicate that resilience may be a key adaptive resource, through which positive leisure experiences can be transformed into stronger efficacy beliefs. This emphasis on PR → SE chain builds a psychologically meaningful bridge between motivation quality and ability related self perception in emerging adulthood (a developmental stage characterized by increased academic and social pressure), advancing SDT based research.

Based on the SDT proposed by Ryan and Deci (52), this study reveals the internal mechanism of exercise motivation in relation to young adults' mental health from the perspective of motivation psychology, which has important theoretical value. First of all, the research expands the application scope of SDT from the traditional path of “motivation-behavior” to the continuous chain of “motivation-emotional experience-psychological resources-ability belief,” confirming that intrinsic motivation is associated with behavioral outcomes but also is associated with improved individual psychological growth through positive emotions and psychological adaptation resources (53). Secondly, by incorporating leisure satisfaction and resilience into SDT, it is found that they play a chain mediating role between EM and SE: the positive leisure experience brought by sports makes the intrinsic motivation emotional and experiential, and resilience is the key bridge to transform this experience into long-term psychological capital. Thirdly, the structural equation model verifies the chain path of “exercise motivation → leisure satisfaction → resilience → self-efficacy,” which provides an empirical basis for “exercise is associated with adaptive functioning by meeting the three essential mental requirements: independence, skill, and connection” (54).

Finally, the study proposes a non-drug psychological promotion idea at the level of adolescent emotional disorder prevention: fostering intrinsic motivation and positive leisure experiences may be associated with greater levels of PR and coping capacity and coping ability, thus broadening the SDT in the field of MH. The explanatory boundary of the field also provides a new theoretical perspective for subsequent positive motivation intervention and psychological construction research.

### 5.2 Practical implications

The results of this study have a direct impact on the psychological health promotion strategies for young people in the university environment, which is a developmental stage characterized by identity formation, academic transition, and increased psychosocial needs. Given that exercise motivation is statistically related to the psychological resources of this population, universities may consider implementing structured, self-supporting exercise plans that comply with the principles of self-determination theory (55).

First, schools can design an 8–12 week elective sports activity plan to provide students with meaningful forms of activity such as team sports, aerobic training, yoga, or outdoor recreation. Such plans may include structured goal setting meetings and regular feedback to support autonomy, competence, and relevance (56). The effectiveness of the project can be evaluated by using validated scales to assess leisure satisfaction, psychological resilience, and self-efficacy before and after intervention.

Second, physical education courses may embed brief reflection modules after exercise, such as guided self-assessment logs or group discussions focused on coping with experiences and emotional reactions. Previous studies have shown that positive exercise related experiences are associated with enhanced psychological resilience and well-being (12, 57). Embedding these structured reflective contents into existing courses is a feasible modification that does not require substantial institutional restructuring.

Third, the university counseling center can collaborate with the Department of Physical Education to develop a “sports based flexible workshop” that combines moderate physical activity with stress management and emotion regulation training (58). The changes in participation rate, retention rate, resilience, and self-efficacy scores can serve as measurable outcome indicators. This interdisciplinary approach may help transform motivational processes into adaptive psychological resources for young people.

Finally, educators can intentionally enhance self-efficacy development in sports environments by providing mastery oriented feedback, simulating coping strategies, and promoting successful task experiences, which have been shown to be associated with improving mental health (59). These structured and assessable methods may help develop sustainable MH promotion frameworks for young people in higher education.

### 5.3 Limitations and future research direction

First, the cross-sectional design precludes conclusions regarding causal relationships or temporal precedence among exercise motivation, PR, and SE. Although the structural model specifies directional paths based on self-determination theory, the data were collected at a single time point, making it impossible to determine whether exercise motivation precedes resilience and self-efficacy or whether reciprocal relationships may exist. Future longitudinal or cross-lagged panel studies are needed to clarify the temporal ordering of these constructs.

Second, although PLS-SEM is suitable for examining complex models with multiple latent variables and exploratory prediction-oriented research, it does not establish causal inference or confirm temporal directionality. The path coefficients reflect statistical associations rather than verified causal mechanisms. Therefore, the proposed model should be interpreted as a theoretically grounded structural representation rather than definitive evidence of causal processes.

Third, the sample comes from a single region and is composed only of college students aged 18–23. Cultural, educational, and social environmental factors may affect motivational processes and psychological resources. Therefore, generalizing research results to other age groups, regions, or cultural environments is limited.

To test stability, future research should use experimental or longitudinal designs and the directionality of proposed pathways. Multi regional sampling and cross-cultural comparison will further enhance the external validity of the model. In addition, incorporating objective behavioral indicators or multi-source data can reduce potential common methodological biases related to self-report measures.

## 6 Conclusion

This study identified statistically significant associations among EM, LS, PR, and SE in young adults. The proposed structural model suggests that experiential and adaptive psychological resources may account for the association between EM and SE within the SDT framework. Given the cross-sectional design, the findings should be interpreted as associative rather than causal. Further longitudinal and experimental research is needed to clarify the temporal dynamics of these relationships.

## Data Availability

The original contributions presented in the study are included in the article/supplementary material, further inquiries can be directed to the corresponding author.
